# A Case of Digoxin Toxicity Due to Acute Renal Failure

**DOI:** 10.7759/cureus.17599

**Published:** 2021-08-31

**Authors:** Stephanie Digiovanni-Kinsley, Brandon Duke, Richard Giovane, Cameron Paisley

**Affiliations:** 1 Family Medicine, University of Alabama, Tuscaloosa, USA; 2 Family Medicine, Regional Medical Center Clinic, Greenville, USA; 3 Internal Medicine, University of Alabama, Tuscaloosa, USA

**Keywords:** digoxin toxicity, ekg changes, renal failure, digoxin toxicity treatment, antibody

## Abstract

Since the publication of the Digitalis Investigation Group trial in 1997, digoxin use has declined significantly. Medications such as angiotensin-converting enzyme inhibitors (ACEi) or angiotensin receptor blockers (ARBs) and beta-blockers that have been demonstrated to have a decrease in morbidity and mortality are prescribed in favor of digoxin. Despite the reduction in digoxin use and improved therapeutic monitoring, digoxin toxicity remains a significant cause of morbidity and mortality. When digoxin toxicity is suspected, patients should be managed with supportive care, including discontinuation of the medication, and consideration for administration of digoxin-specific antibody fragment. We present a case of digoxin toxicity precipitated by acute renal failure, with a discussion on the pathophysiology and diagnosis of digoxin toxicity, along with the indications for administration of digoxin-specific antibody fragments. While digoxin toxicity is prescribed less commonly, physicians need to maintain a high index of suspicion and be comfortable with administering digoxin-specific antibody fragment in these scenarios.

## Introduction

The medical benefits of digoxin were first described by William Withering in 1785, since then it has been an integral part of clinical practice for over 200 years. Digoxin is currently used in the treatment of chronic systolic and diastolic heart failure, as well as atrial fibrillation [1,2]. Since the publication of the Digitalis Investigation Group trial in 1997, digoxin use in the treatment of patients with symptomatic chronic heart failure with normal sinus rhythm and left ventricular dysfunction has declined from an estimated 80% to less than 30%, as the evidence in support of angiotensin-converting enzyme inhibitors (ACEi), angiotensin receptor blockers (ARBs) and beta blockers have grown in favor of decreasing significant patient mortality and morbidity [1,3]. Current guidelines on the diagnosis and treatment of heart failure from both the European Society for Cardiology and the American College of Cardiology Foundation/American Heart Association Task Force recommend limiting digoxin use for patients with continued heart failure symptoms despite optimal therapy with ACEI, ARBs, beta-blockers, and mineralocorticoid receptor antagonists [4,5]. Digoxin use is also recommended in patients with atrial fibrillation with rapid ventricular response despite treatment with other agents, or when other treatments cannot be used [4,5].

Despite significant reduction in digoxin use, improved therapeutic monitoring, and the availability of digoxin-Fab, digoxin toxicity remains a significant cause of hospitalizations due to adverse drug events, morbidity, and mortality [6]. From 2005 to 2010, digoxin toxicity accounted for over 5,000 annual emergency department (ED) visits in the U.S., amounting to 1.0% of all ED visits for adverse drug events (ADEs), and over 75% of these visits resulted in hospitalization [7]. The rate of hospitalization due to digoxin toxicity is far higher than the estimated 20%-50% due to other drugs commonly implicated in ED visits for ADEs [8]. Physicians need to be knowledgeable of the clinical signs and symptoms of digoxin toxicity, as well as how to distinguish electrocardiographic evidence of the digoxin effect (scooped ST-segments), digoxin excess (atrioventricular block), and digoxin toxicity (ventricular arrhythmias and hyperkalemia) [9,10]. We present a case of digoxin toxicity precipitated by acute renal failure with a discussion of the pathophysiology and diagnosis of digoxin toxicity, and indications for administration of digoxin-specific antibody fragments.

## Case presentation

A 62-year-old Caucasian male with a past medical history of systolic heart failure with ejection fraction (HFrEF) of less than 30%, chronic obstructive pulmonary disease (COPD) who is on 3L of supplemental oxygen, atrial fibrillation, coronary artery disease, and hypertension was evaluated in the ED for acute onset shortness of breath at rest. The patient reported increased shortness of breath that started immediately prior to arrival while he was seated at home at rest with no relief after a trial of albuterol and steroid inhalers. He stated that he had decreased oral intake for the past week, due to pain in his tongue and decreased appetite. He denied cough, fever, chest pain, vision changes, headache, lower extremity edema, recent travel, or sick contacts. The patient lives at home with his wife and reports taking lisinopril, sacubitril/valsartan, furosemide, metoprolol 25 mg BID, and digoxin 0.125 mg BID. Ten days prior to this presentation, he was discharged from the hospital after treatment for a COPD exacerbation. He did have a prior smoking history, but quit approximately 20 months ago; he consumed one beer per day and denied illicit drug use. At his baseline, he ambulates without assistance. 

**Figure 1:**
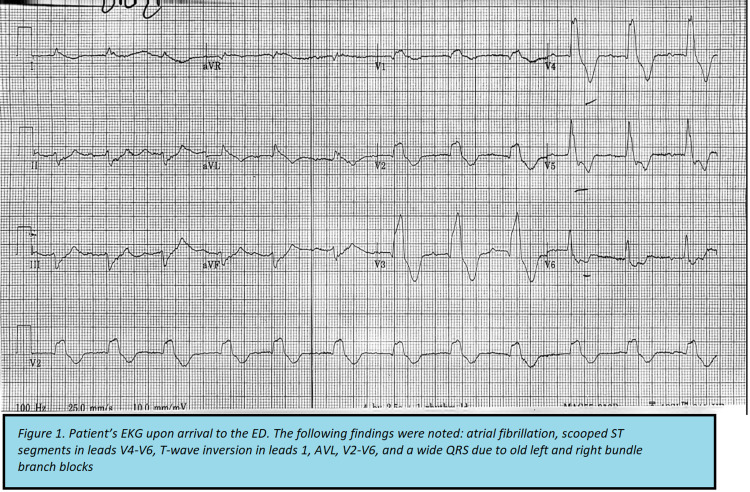
EKG of the patient upon arrival to the emergency department

The patient’s vital signs were: blood pressure 96/63 mmHg, pulse 103 bpm, respiration rate 25 breaths/min, rectal temperature 96.5 degrees Fahrenheit, and oxygen saturation of 88% with 3L of supplemental oxygen via nasal cannula. The patient appeared chronically ill, thin, and in respiratory distress with tachypnea and accessory muscle use. He had dry mucosa and glossitis. His heart rate was irregularly irregular without audible murmurs. He had mild bilateral lower extremity edema and his skin was dry and cool to the touch without cyanosis. His physical exam was otherwise unremarkable. A complete metabolic panel was consistent with acute renal failure demonstrated by hyperkalemia with a potassium level of 8.1 mmol/L, hyperphosphatemia with phosphorus level of 12.7 mg/dL, and hypermagnesemia with a magnesium level of 3.5 mg/dL, elevated blood urea nitrogen of 124 mg/dL, and serum creatinine level of 3.84 mg/dL. Complete blood count was significant for leukocytosis with white blood cell count of 16,460 /uL, and anemia with hemoglobin level of 9.0 g/dL and hematocrit of 28.2%. N-terminal pro-B-type natriuretic peptide was markedly elevated at 5647 pg/mL and digoxin level was elevated as well at 2.65 ng/mL. Arterial blood gas with supplemental oxygen, high-sensitivity troponin, thyroid-stimulating hormone, cortisol level, and urinalysis were all unremarkable. Electrocardiogram (EKG) upon arrival to the ED was remarkable for atrial fibrillation at a rate of 103 beats per minute, scooped ST segments in leads V4-V6, T-wave inversion in leads 1, AVL, V2-V6, and a wide QRS due to old left and right bundle branch blocks (Figure 1). Chest X-ray in the ED was unremarkable.

The patient was diagnosed with cardiogenic shock due to his clinical appearance on physical exam, unstable vital signs, and lab work. This was exacerbated by an underlying acute renal failure due to poor oral intake and nephrotoxicity from his medications. He was given two liters of 0.9% saline; without improvement in blood pressure, and he required vasopressor support with norepinephrine infusion titrated to a mean arterial pressure (MAP) of at least 65 mmHg. Hyperkalemia was treated with two administrations each of 25 mg dextrose and 10 units of insulin but did not improve significantly. Two vials of digoxin antibody fragment were administered in the ED. Upon transfer to the intensive care unit (ICU), all nephrotoxic medications were discontinued, and intravenous fluids were changed to a continuous bicarbonate infusion (0.45% saline plus 100 mEq/L bicarbonate) at 150 cc/hr. The patient’s MAP remained suboptimal overnight despite maximum dose norepinephrine, and vasopressin was added, along with placement of a central venous catheter. Cardiology, nephrology, and pulmonology were consulted. Per nephrology, dialysis was not indicated as although the patient's potassium was critical, they wanted to continue medications to lower his potassium as well as stating that dialysis would not lower his serum digoxin and therefore dialysis could be deferred.

On his second hospital day, the patient appeared clinically improved. He tolerated decreasing his oxygen supplementation from four to three liters via nasal cannula. Vasopressor medications were discontinued, and intravenous fluids were changed to 0.9% saline. His serum electrolytes normalized. Also, his renal function improved with serum creatinine decreased to 1.99 mg/dL. Cardiology, nephrology, and pulmonology specialists agreed with our diagnosis of digoxin toxicity and current management. His discharge out of the ICU was delayed due to a gastrointestinal bleed secondary to chronic gastritis, causing a decrease in hemoglobin from 7.7 g/dL to 6.5 g/dL. The patient was transfused one unit of packed red blood cells, started on intravenous pantoprazole 80 mg twice daily, and transferred to the general medical floor on his third hospital day. His heart failure medications were slowly resumed with assistance from our cardiology specialist, and he remained improved and stable throughout the remainder of his hospital stay. He required additional hospital evaluation and management for gastrointestinal bleeding, and was discharged to his home on his ninth hospital day.

## Discussion

Digoxin exerts inotropic effects on the heart through inhibition of the sodium-potassium adenosine triphosphatase (Na-K ATPase). Inhibition of the Na-K ATPase increases intracellular sodium concentration and extracellular potassium concentration. The resulting loss of the transmembrane sodium gradient decreases the activity of the sodium-calcium (Na/Ca) exchanger. Decreased activity of the Na/Ca exchanger increases intracellular calcium levels, triggering calcium release from the sarcoplasmic reticulum, increasing myocyte contractile force [2,9,11]. In patients with systolic dysfunction, digoxin increases cardiac output by improving left ventricular ejection fraction, without increasing the heart rate or lowering blood pressure [9]. Digoxin also functions as a neurohormonal modulator through its effects on the Na-K ATPase in extra-cardiac tissues. Epinephrine and renin concentrations are decreased through sympatholytic effects by digoxin-mediated increased vagal tone. This increased parasympathetic tone also improves carotid sinus baroreceptor sensitivity [1,9,11].

At therapeutic levels, digoxin slows firing through the sinoatrial node, prolongs conduction at the atrioventricular node and decreases automaticity. At toxic levels, digoxin-mediated increased myocyte excitability due to decreased resting membrane potential combined with increased vagal tone can cause almost any type of cardiac arrhythmias [1,2]. Digoxin has little effect on the conduction velocity, consequently, bundle branch blocks and intraventricular conduction delays are rarely due to digoxin toxicity [1]. The most common electrocardiographic manifestations of toxicity include atrioventricular block, escape rhythms, and premature ventricular contractions [1,11]. These common arrhythmias are nonspecific, with many etiologies, but junctional tachycardia and bi-ventricular tachycardia are strongly suggestive of digoxin toxicity [2,10].

Most patients that present with digoxin toxicity are elderly, have other cardiac and noncardiac comorbidities, and take multiple medications including those known to potentially interfere with serum digoxin concentrations (SDC) [12]. The most common presentation of digoxin toxicity is due to unintentional overdose or decreased drug clearance due to hepatic or renal dysfunction, as opposed to acute, intentional ingestions [2,6,12]. Most patients with acute toxicity present with anorexia, nausea, emesis, and fatigue. Chronic toxicity usually presents with an insidious onset over days to weeks, of neurologic symptoms including confusion, disorientation, and fatigue. Visual changes such as halos, scotomas, flashing lights, and xanthopsia may also be reported [2,11]. Historically, bradycardia has been associated with digoxin toxicity, but recent studies have suggested that bradycardia in the setting of digoxin toxicity is more likely multifactorial and minimally improves with treatment [6,11]. Renal failure is the most common precipitating event to digoxin toxicity causing decreased drug clearance, and is associated with worse outcomes when concurrent hyperkalemia and ventricular arrhythmias are present, as in our patient [13,14]. Chronic digoxin toxicity commonly occurs with electrolyte disorders of potassium, magnesium, and calcium due to the coadministration of diuretics in this patient population [13]. SDC should not be used to diagnose digoxin toxicity, as toxicity can occur at elevated levels (>2.0 ng/mL), or at therapeutic levels (0.5-0.9 ng/mL) with concurrent metabolic disturbance [1,11].

**Figure 2:**
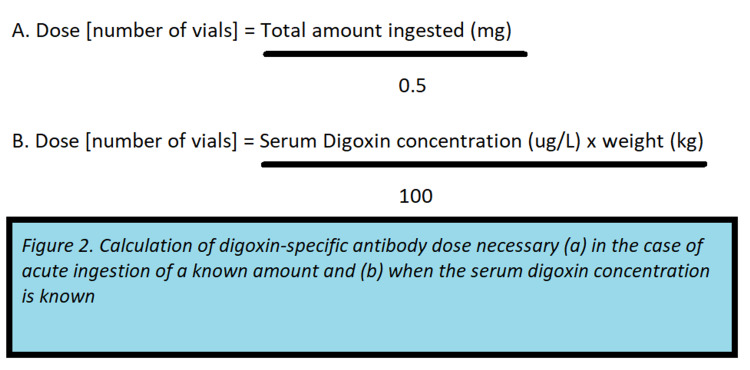
Calculation of digoxin-specific antibody dose necessary

All patients presenting with or suspected of digoxin toxicity should be managed with supportive care, including discontinuation of the medication [10]. Patients with chronic digoxin toxicity presenting with elevated SDC commonly have multiple acute comorbidities including dehydration and acute renal failure, and supportive care along with management of these comorbidities may have a more significant impact on clinical outcomes than the administration of digoxin-specific antibody [6,12]. Although patients with renal failure and hyperkalemia may meet the criteria for emergent hemodialysis, this is ineffective in digoxin toxicity due to the extensive fat distribution of the drug [1]. Indications for treatment with digoxin-specific antibody in the setting of chronic toxicity include life-threatening arrhythmias (ventricular tachycardia or fibrillation), severe bradycardia unresponsive to atropine, impaired renal function with an increased digoxin concentration, and hyperkalemia resistant to standard potassium-lowering treatments [6,10]. Two methods are widely accepted to determine the necessary dose of digoxin-specific antibody, the first method requires knowledge of the SDC, and the second method is intended for use when the amount ingested is known (Figure 2) [1].

Observational studies on clinical outcomes in patients treated with digoxin-specific antibody have suggested that it does not significantly improve mortality or length of stay. These studies, however, excluded patients with ventricular arrhythmias and endorsed the current guidelines for digoxin-specific antibody treatment [6,12].

Prior to the publication of the Digitalis Investigation Group trial, digoxin was reported to be prescribed to over 80% of patients for the treatment of heart failure. Digoxin use has declined over the past 20 years, in part due to concerns about safety after recent observational studies have suggested increased mortality in patients prescribed digoxin [14,15]. Despite the controversy about the safety of digoxin, its use remains a significant cause for ED visits and hospitalizations for adverse drug events [7,9].

## Conclusions

Careful patient selection with consideration of individual risk factors, serum monitoring, and recognition of digoxin-mediated arrhythmias and clinical manifestations remain paramount in the effective treatment and decreasing morbidity and mortality among this patient population.
